# Personalized Management and Timing of Thymectomy in Juvenile Myasthenia Gravis: Insights from Routine Clinical Scale Use in a Single-Center Retrospective Cohort—A Case Series

**DOI:** 10.3390/children12101389

**Published:** 2025-10-15

**Authors:** Gulten Ozturk, Olcay Unver, Elif Acar Arslan, Nezih Onur Ermerak, Bilgihan Bıkmazer, Hakkı Akbeyaz, Burcu Karakayali, Sermin Aksoy Ozcan, Gulcan Akyuz, Pınar Ergenekon, Yasemin Gokdemir, Ela Erdem Eralp, Pınar Kahraman Koytak, Kayıhan Uluc, Dilsad Turkdogan

**Affiliations:** 1 Department of Pediatric Neurology, Marmara University Pendik Education and Research Hospital, Istanbul 34899, Turkey; olcaymd@hotmail.com (O.U.); elifacararslan@gmail.com (E.A.A.); dr.bilgihan@hotmail.com (B.B.); hakkiakbeyaz@gmail.com (H.A.); burcukarakayali@hotmail.com (B.K.); serminaksoy@yahoo.com (S.A.O.); gulcan.akyuz@hotmail.com (G.A.); dturkdogan@hotmail.com (D.T.); 2 Department of Thoracic Surgery, Marmara University Pendik Education and Research Hospital, Istanbul 34899, Turkey; onur.ermerak@marmara.edu.tr; 3 Department of Pediatric Pulmonology, Marmara University Pendik Education and Research Hospital, Istanbul 34899, Turkey; drpergenekon@hotmail.com (P.E.); yasemingokdemir@yahoo.com.tr (Y.G.); elaerdem@yahoo.com (E.E.E.); 4 Department of Neurology, Marmara University Pendik Education and Research Hospital, Istanbul 34899, Turkey; pinarkahraman@yahoo.com (P.K.K.); kayihanu@yahoo.com (K.U.)

**Keywords:** juvenile myasthenia gravis, Quantitative Myasthenia Gravis Score, PM-QOL15, thymectomy, intravenous immunoglobulin, corticosteroids, azathioprine, pediatric neuromuscular disorders

## Abstract

**Introduction:** Juvenile myasthenia gravis (JMG) is a rare autoimmune disorder with a variable clinical course and limited pediatric-specific treatment guidelines. Objective clinical scales, such as the Quantitative Myasthenia Gravis (QMG) Score and the Pediatric Myasthenia Gravis Quality of Life 15 (PM-QOL15), may support individualized management, but their role in routine practice remains underexplored. **Methods:** We retrospectively reviewed 10 seropositive JMG patients followed at a single tertiary neuromuscular clinic between 2014 and 2024. All patients underwent a systematic assessment with QMG at each visit, while PM-QOL15 was administered at the final visit. Clinical data, comorbidities, antibody status, treatment modalities, and outcomes were analyzed. Associations between treatment strategies, comorbidities, and scale scores were explored using appropriate statistical methods. **Results:** Seven patients (70%) underwent thymectomy, resulting in a reduction in mean QMG scores from 7.7 to 2.4, though residual relapses were observed. Chronic intravenous immunoglobulin (IVIG) therapy, administered to 70% of patients, did not significantly reduce relapse rates or steroid exposure and was associated with higher QMG scores in the second year, suggesting use in more severe phenotypes rather than therapeutic efficacy. Prolonged corticosteroid therapy did not improve remission time or relapse frequency and was complicated by major adverse effects in two patients. Timing of azathioprine initiation showed no significant correlation with relapse frequency. PM-QOL15 correlated strongly with mean QMG (r = 0.88, *p* < 0.001), reflecting cumulative disease burden. Patients with comorbidities required longer stabilization, although differences were not statistically significant. **Conclusions:** The routine integration of QMG and PM-QOL15 into follow-up may facilitate the earlier recognition of subclinical deterioration, provide objective measures of treatment response, and guide personalized management in JMG. Thymectomy showed benefit in selected patients, while the long-term roles of IVIG and corticosteroids remain uncertain. Larger multicenter prospective studies are warranted to confirm these findings and refine evidence-based strategies for pediatric JMG.

## 1. Introduction

Juvenile myasthenia gravis (JMG) is a rare autoimmune neuromuscular disorder that presents with clinical and immunological characteristics distinct from the adult form of myasthenia gravis [1,2]. Its management remains challenging because of the heterogeneous disease course and the relatively high frequency of spontaneous remission in children, which complicates early therapeutic decision-making. While conservative approaches may increase the risk of disease progression and long-term morbidity, aggressive immunosuppressive strategies can cause considerable adverse effects in the pediatric population [3].

Although the number of studies focusing on JMG has increased in recent years, universally accepted guidelines for standardized treatment protocols and the optimal timing of thymectomy are still lacking. Current treatment strategies are largely informed by institutional experiences and small case series rather than robust randomized controlled trials [4,5]. Increasing evidence supports thymectomy as an effective and safe therapeutic option for children with JMG; however, the rarity of the disease means most available studies are multicenter in design, and large, single-center series remain scarce [6,7]. Expert opinion and observational data suggest that the greatest benefit occurs when thymectomy is performed within the first year of symptom onset, but no consensus or standardized recommendations currently exist regarding the timing of surgery in pediatric patients [8,9].

Objective clinical instruments such as the Quantitative Myasthenia Gravis Score (QMGS), the Myasthenia Gravis Activities of Daily Living (MG-ADL), and the Pediatric Myasthenia Gravis Quality of Life 15 (PM-QOL15) scale provide valuable means of assessing disease severity and treatment response. These tools are particularly beneficial in pediatric populations, where accurate symptom reporting may be inconsistent or limited by developmental communication barriers [10]. Despite their potential advantages, their use in routine clinical practice remains limited, largely due to time constraints, the complexity of administration, and the limited availability of pediatric-specific validation [11].

This is a retrospective observational study that focuses on the follow-up data of seropositive JMG patients who were systematically monitored with the QMGS in addition to neurological examination at every visit. All assessments were performed by the same pediatric neurologist, ensuring consistency in both clinical and scale-based evaluations. This approach enabled us to explore the role of routine structured assessments in guiding individualized treatment strategies, including decisions regarding the timing of thymectomy. Clinical outcomes, including medication duration and treatment responses, are presented and correlated with QMG scores.

Our findings aim to underscore the practical benefits of incorporating standardized clinical scales into everyday pediatric neuromuscular care and to provide preliminary evidence supporting their utility in optimizing therapeutic decision-making for JMG.

## 2. Materials and Methods

### 2.1. Participants

Medical records of all patients diagnosed with JMG and followed at our neuromuscular clinic between May 2014 and May 2024 were retrospectively analyzed. Seropositive patients with generalized JMG who remained in follow-up for at least 2 years and had QMG scoring performed routinely at every follow-up visit were included in the study. Patients under 6 years of age at initial visit and/or those who were not compliant with spirometry were excluded. Seronegative patients were excluded because of the diversity in diagnostic tests performed for differential diagnosis and the heterogeneity in their treatment protocols.

### 2.2. Methods

Patient demographic data, including sex, age at disease onset, clinical severity, serologic antibody status, treatment modalities, treatment-related side effects, and long-term follow-up outcomes, were extracted from medical records. Detailed data of patients who had undergone thymectomy were analyzed and reported independently. The indications for thymectomy, time from diagnosis to surgery, and clinical characteristics of the patients were noted.

Concomitant comorbidities of the patients were recorded. According to the follow-up protocol of the clinic, the screened tests for concomitant autoimmunity were antibodies against thyroglobulin, thyroid peroxidase, dsDNA, tissue transglutaminase IgA, tissue transglutaminase IgG, gliadin IgG, gliadin IgA, phosphatidylserine IgA and IgG, cardiolipin IgM and IgG, and neutrophil cytoplasmic antibodies (ANCA) in all patients. Additionally, anti-nuclear antibody (ANA), antibodies to glutamic acid decarboxylase (anti-GAD), anti-liver-kidney microsomal antibodies, anti-parietal antibodies, and rheumatoid factor antibodies (anti-RF) were screened in some patients, regardless of any relevant symptoms. Thoracic magnetic resonance imaging was performed on all patients. Those aged > 6 years were referred for spirometry testing.

Clinical status at initial evaluation was classified using the Myasthenia Gravis Foundation of America (MGFA) classification. According to this classification, ‘0′ points were assigned to asymptomatic patients, ‘1′ point for ocular involvement, ‘2′ points for mild muscle weakness, ‘3’ points for moderate weakness, and ‘4’ points for severe muscle weakness; each of the muscle weakness occurring with or without ocular involvement, and ‘5’ for respiratory insufficiency requiring intubation. Axial and extremity involvement were subclassified as ‘a,’ and accompanying bulbar involvement was subclassified as ‘b.’

At each visit, the QMGS was performed in all patients aged > 6 years. Hand-held dynamometry was performed twice at each scoring, and the mean of these two scores was noted for each hand. Spirometry was performed on the patients by a qualified nurse in the pediatric pulmonology unit and included in the scoring of QMGS.

The Pediatric Myasthenia Gravis Quality of Life 15 (PM-QOL15) scale was administered once to all patients during their last visit with the aim of reflecting their present clinical status from their own perspective [12]. For patients under 10 years of age, parental feedback was used to fill out the PM-QOL15 scale.

Individual follow-up graphics were created for the patients, including their QMGS at each visit, current treatment, and clinical status. Canva Pro (http://www.canva.com, accessed on 25 August 2025) was used to create individual graphics. Statistical analysis was performed to compare treatment modalities and clinical outcomes.

### 2.3. Treatment Protocol of the Clinic

Pyridostigmine was initiated in all patients upon presentation, gradually increasing based on the clinical status of the patient. Corticosteroids were started in patients with generalized myasthenia either orally or intravenously, depending on clinical severity. Intravenous methylprednisolone was used in rare cases of myasthenic crisis with severe bulbar and respiratory symptoms, at varying doses, with a maximum dose of 1 g/d in patients admitted to the intensive care unit.

Steroid use of the patients was categorized as middle/high dose if used at a dose higher than 0.5 mg/kg/d. Intravenous immunoglobulin (IVIG) was also concomitantly administered to patients who presented with bulbar symptoms during their hospital stay. Monthly IVIG therapy as maintenance was preferred either in patients with frequent clinical relapses or in those for whom steroid treatment was not feasible due to adverse effects or refusal to use corticosteroids. The treatment regimen was planned as a loading dose of 2 gr/kg followed by monthly doses of 1 gr/kg.

Azathioprine was the preferred steroid-sparing immunomodulating agent for patients whose steroid dosages could not be reduced to target doses after 3 months of treatment. The initial dose for azathioprine was 0.5 mg/kg/d, with a maximum dose of 2 mg/kg/d in our cohort. A clinical attack was defined as clinical deterioration of the patients with subjective complaints and/or physical examination findings.

### 2.4. Statistics

Statistical analyses were performed using the SPSS software, version 27.0. The normality of the variables was assessed through histogram plots and the Kolmogorov–Smirnov test. Descriptive analyses were presented using mean, standard deviation, median, and minimum–maximum values. For comparisons between two groups, the Independent-Samples T-Test was applied to variables with a normal distribution (parametric). The Mann–Whitney U test was used for correlations. A *p*-value of less than 0.05 was considered statistically significant. A simple linear regression test was used for correlation analysis.

### 2.5. Ethics

Ethics approval for the study was obtained from the local ethics committee (approved on 20 September 2024, 09.2024.957).

## 3. Results

### 3.1. General Characteristics

The study sample consisted of 10 patients, of whom 6 (60.0%) were female and 4 (40.0%) were male. The mean age at diagnosis was 153.1 ± 31.86 months, with a median of 149.5 months (range: 111–204). Five patients were diagnosed at post-pubertal age (50%). The mean follow-up time was 50.5 ± 14.77 months, and the median follow-up duration was 48 months (range: 24–76). The mean age at thymectomy was 167.71 ± 38.91 months, with a median of 168 months (range: 123–229). The mean PM-QOL15 score of the cohort at the last visit was 12.3 ± 17.78, with a median of 2.5 (range: 0–54). In patients with higher scores, the highest points were concentrated on questions regarding the adverse effects of the chronicity of the disease on quality of life and psychology.

Autoimmune antibodies, apart from myasthenia-associated antibodies, were found in seven of the 10 patients, with one patient showing double positivity (ANA (+) in 5 patients; Anti ds DNA in one patient; Anti GAD in one patient, and Anti-parietal Ab in one patient). One patient was diagnosed with a clinically significant additional autoimmune disease (growth hormone deficiency, immune thrombocytopenic purpura (ITP), and systemic lupus erythematosus (SLE)). One patient had concomitant absence epilepsy resistant to treatment, and one patient was diagnosed with severe anxiety disorder.

At the initial presentation, patients were classified according to the MGFA classification: Class 1 (two patients), Class 2A (one patient), Class 3A (two patients), Class 3B (two patients), and Class 4B (three patients). Four patients presented with bulbar symptoms (nasal speech and difficulty in swallowing), three patients had ptosis, two patients had ophthalmoparesis, and two presented with tiredness. Table 1 shows the demographic and clinical characteristics of the study cohort.

Two major corticosteroid complications observed in our cohort that required discontinuation were hip fracture in one patient and bilateral cataracts in another. The hip fracture occurred within the first month of treatment when the patient was taking prednisolone (1 mg/kg/d). These patients were administered monthly IVIG and were referred for thymectomy.

Regarding disease severity, the mean basal QMG score for the whole group was 12.2 ± 4.39, with a median of 13 (range: 3–17). In terms of longitudinal disease course, the median QMG scores progressively declined over time, with a mean of 6.87 ± 4.21 in the first year (median 5.8; range: 1.6–13), 3.31 ± 2.26 in the second year (median 2.6; range: 0–7.5), and 2.23 ± 3.1 in the third year (median 1.15; range: 0–9.5). The mean total duration of steroid use was 7.1 ± 10.24 months (median 2; range: 0–26). The mean time to initiation of azathioprine (AZP) treatment was 5.14 ± 4.14 months, with a median of 4 months (range: 1–12).

### 3.2. Thymectomy Data

Thymectomy was performed on 7 patients (70%). The two main reasons for thymectomy were steroid dependence and resistance to treatment, except for one who had a thymoma. Pathological evaluation revealed thymic hyperplasia in four patients, a thymoma in one patient, and a normal thymus in one patient. Video-assisted thoracoscopic surgery was performed in all patients, and the mean surgery time was 132.5 ± 24.03 min. Table 2 shows the clinical characteristics of thymectomy patients.

Patients experienced a mean of 1.7 ± 1.57 attacks overall, including 1.29 ± 0.95 attacks before thymectomy and 0.71 ± 1.25 attacks after thymectomy.

The mean QMG score prior to thymectomy was 7.71 ± 4.39 (median 7; range: 2–14), while the mean score decreased to 2.43 ± 2.3 after thymectomy (median 2; range: 0–6). The mean time from diagnosis to thymectomy was 7.5 ± 7.92 months, with a median of 6 months (range: 0–23). Follow-up characteristics of thymectomy patients are shown in Table 3.

Regarding the entire cohort, 70.0% of the patients received monthly IVIG treatment, and 70.0% were treated with azathioprine (AZP). Comorbid diseases were present in 30.0% of the patients, and steroid-related complications or side effects were observed in 20% of the study population (50.0%). In addition, 60.0% of the patients required middle- to high-dose steroid therapy for more than three months.

### 3.3. Maintenance IVIG Use

In the first year, patients without monthly IVIG had a mean median QMG score of 4.87 ± 1.62 (median 5.8; range: 3–5.8), whereas those with IVIG had a higher mean of 7.73 ± 4.78 (median 6.8; range: 1.6–13). This difference was not statistically significant (*p* = 0.197).

In the second year, patients without IVIG demonstrated a mean score of 1.23 ± 1.12 (median 1.5; range: 0–2.2), while patients receiving IVIG showed a higher mean score of 4.2 ± 2.04 (median 3.6; range: 2–7.5). This difference reached statistical significance (*p* = 0.049) (Table 4).

Patients who did not receive monthly IVIG experienced a mean of 1.0 ± 0 attacks, with a median of 1 (range: 1–1), while those who received IVIG had a mean of 2.0 ± 1.83 attacks, with a median of 2 (range: 0–5). This difference was not statistically significant (*p* = 0.197). Mean basal QMG score of patients on monthly IVIG was 12.2 ± 4.3, with a median of 13 (3–17), and mean basal QMG score of patients who did not get monthly IVIG was 12.3 ± 4.8, with a median of 13.5 (11–17). There was no statistical difference between the two groups (*p* > 0.05).

Before thymectomy, the mean number of attacks was 1.0 ± 0 (median 1; range: 1–1) in the non-IVIG group and 1.4 ± 1.14 (median 1; range: 0–3) in the IVIG group, with no significant difference between the groups (*p* = 0.659).

After thymectomy, patients without IVIG had no attacks (mean 0.0 ± 0; median 0; range: 0–0), whereas those receiving IVIG had a mean of 1.0 ± 1.41 attacks, with a median of 0 (range: 0–3). The difference did not reach statistical significance (*p* = 0.189) (Independent-Samples T-Test). Additional antibody positivity has not been found statistically related to the number of attacks or the mean time until symptom-free status (*p* > 0.05; Independent-Samples T-Test).

### 3.4. Steroid Use

Patients who did not receive middle- or high-dose steroids for more than three months had a mean time to symptom-free status of 5.5 ± 8.35 months (median 1.5; range: 1–18), while those with prolonged steroid use had a mean of 4.0 ± 1.67 months (median 3.5; range: 2–6). This difference was not statistically significant (*p* = 0.745).

The mean number of attacks was higher in the group without prolonged steroid use (2.5 ± 1.91; median 2; range: 1–5) compared to those with prolonged use (1.17 ± 1.17; median 1; range: 0–3), although the difference did not reach statistical significance (*p* = 0.204) (Table 5).

Before thymectomy, patients without prolonged steroid therapy experienced more attacks (mean 2.0 ± 1.0; median 2; range: 1–3) compared with those on longer steroid therapy (mean 0.75 ± 0.5; median 1; range: 0–1), and this difference showed a trend toward significance but was not statistically significant (*p* = 0.078).

After thymectomy, the mean number of attacks was similar between groups (1.0 ± 1.73 vs. 0.5 ± 1.0), with no significant difference (*p* = 0.646).

Patients who did not receive steroids for more than three months had a mean median QMG score of 5.34 ± 3.74 in the first year (median 5.2; range: 1.6–13), whereas those with prolonged steroid use had a higher score of 10.43 ± 3.23 (median 11.5; range: 6.8–13). Although this difference suggested a trend toward higher QMG scores in the prolonged steroid group, it did not reach statistical significance (*p* = 0.076).

In the second year, the mean median QMG score was 2.91 ± 2.09 (median 2.2; range: 0–5.6) in the non-prolonged group and 4.23 ± 2.83 (median 2.7; range: 2.5–7.5) in the prolonged group, with no significant difference (*p* = 0.430).

Regarding disease relapses after thymectomy, patients without prolonged steroid use experienced fewer attacks (mean 0.5 ± 1.0; median 0; range: 0–2) compared with those on prolonged therapy (mean 1.0 ± 1.73; median 0; range: 0–3), but this difference was not statistically significant (*p* = 0.646) (Table 6).

### 3.5. AZP Use

In linear regression analysis, the timing of AZP initiation was not significantly associated with the number of clinical attacks. Each one-month delay in AZP initiation was associated with a non-significant decrease of 0.1 attacks (95% CI: –0.55 to 0.35, *p* = 0.60). The model explained only 6% of the variance in attack frequency (R^2^ = 0.06)

**Figure 1:**
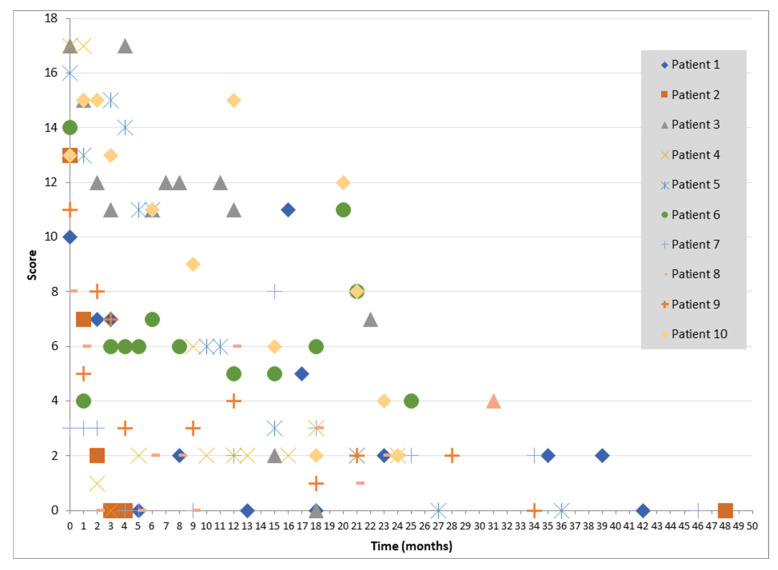
QMG scores of patients at outpatient visits when they reported being symptom-free.

In addition to the expected increase in scores consistent with the patient’s clinical deterioration, isolated increases in QMG scores were also observed in patients who were reported to be asymptomatic (Figure 1).

Figure 1 shows the changes in QMG scores of patients who were reported to be asymptomatic at follow-up visits.

**Figure 2:**
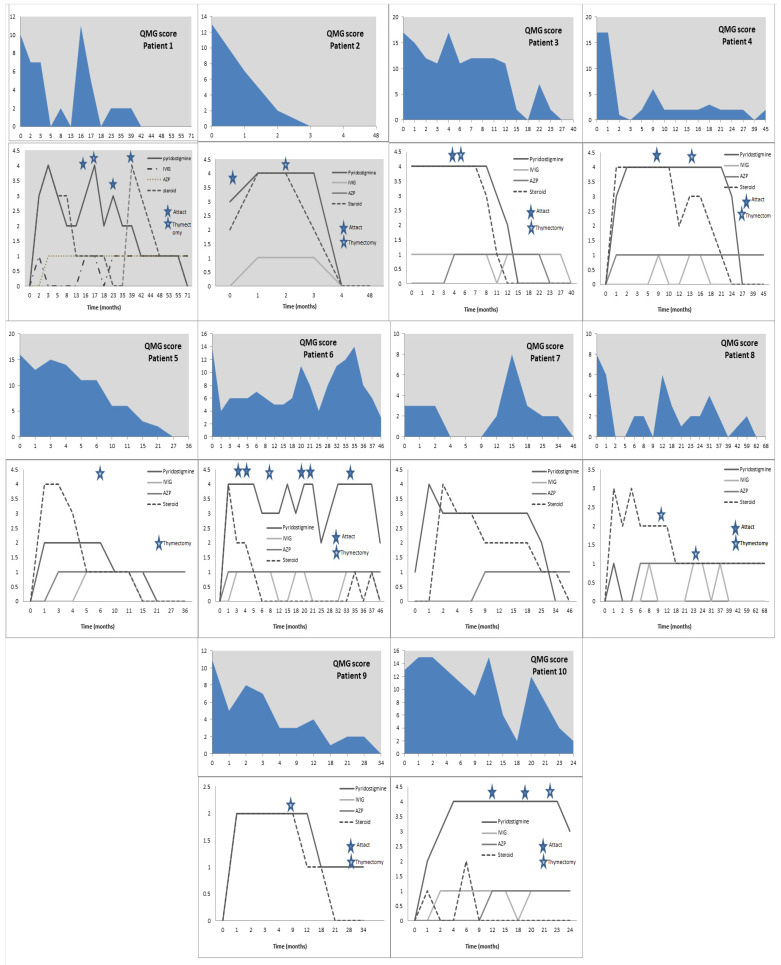
Illustration of individual treatment and follow-up profiles of all patients included in the study cohort, correlating disease-related events (such as clinical attacks and timing of thymectomy) with treatment modifications, dosing adjustments, and QMG scores.

Graph analysis of Figure 2 indicates that QMG score elevations prompted treatment adjustments, even in the absence of reported symptoms, and tapering was deferred until score reductions were achieved. In some cases, abrupt score increases led to treatment intensification before clinical worsening was reported. Among patients transitioned to monthly IVIG due to rising scores and clinical decline, inadequate control necessitated re-initiation of steroids despite adverse effects.

### 3.6. Comorbidities

Patients without comorbidities achieved symptom-free status in a mean of 2.57 ± 1.72 months (median 2; range: 1–6), whereas those with comorbidities required a longer period, with a mean of 9.33 ± 7.57 months (median 6; range: 4–18). This difference did not reach statistical significance (*p* = 0.261).

The mean number of attacks was 1.86 ± 1.68 (median 1; range: 0–5) in patients without comorbidities and 1.33 ± 1.53 (median 1; range: 0–3) in those with comorbidities (*p* = 0.656). Before thymectomy, patients with comorbid diseases experienced more frequent attacks (mean 2.0 ± 1.41; median 2; range: 1–3) compared to those without comorbidities (mean 1.0 ± 0.71; median 1; range: 0–2), although this difference was not statistically significant (*p* = 0.239).

Concomitant presence of antibody status was not significantly associated with time to clinical stabilization (Mann–Whitney U = 7.5, *p* = 0.56) or number of clinical attacks (Mann–Whitney U = 12.5, *p* = 0.72). However, Patient 3, who had intractable absence epilepsy, and Patient 10, who had concomitant severe anxiety disorder, showed higher QMG scores with longer time until clinical stabilization (Figure 2).

### 3.7. PM-QOL15 Scores

Correlation analysis revealed a strong positive association between PM-QOL15 and mean QMG scores (Pearson r = 0.88, *p* = 0.0007; Spearman ρ = 0.81, *p* = 0.0049), while the correlation with the last QMG scores was moderate and did not reach statistical significance (Pearson r = 0.52, *p* = 0.12; Spearman ρ = 0.58, *p* = 0.079)

## 4. Discussion

This study aimed to evaluate the impact of disease-specific scale use in the routine clinical practice of JMG patients on personal treatment strategies and clinical outcomes. Retrospective data of a small cohort diagnosed with seropositive JMG and closely followed by a single neuromuscular clinic were presented.

The findings of this study suggest that incorporating the QMG scale into routine outpatient follow-up of pediatric MG patients over 6 years of age may facilitate the early detection of clinical deterioration before symptom recognition and provide more objective measures for evaluating treatment responses. PM-QOL15, which was used in the study to capture patients’ self-perceived physical and psychological status at the time of evaluation, appeared to reflect the cumulative impact of the disease over time rather than only the most recent clinical state. Although studies in the literature support the usefulness of the PM-QOL15 scale in juvenile myasthenia, no prior work was identified that specifically highlighted this finding [13]. The combined use of QMGS and PM-QOL15 scales in our study allowed the discrimination of psychological deterioration from an increase in muscle weakness and fatigue, thus preventing the patient from being overtreated.

In this cohort of juvenile myasthenia gravis (JMG) patients, a relatively high proportion underwent thymectomy and received maintenance IVIG therapy, which distinguishes this group from other published series. Consistent with prior literature, thymectomy was associated with a substantial reduction in QMG scores, decreasing from a mean of 7.7 before surgery to 2.4 after surgery, supporting its beneficial role in disease control in carefully selected patients [14,15]. Nevertheless, the presence of residual attacks after thymectomy in some patients emphasizes the variability of outcomes and the need for individualized treatment strategies.

Although IVIG is widely used in both acute and maintenance regimens for pediatric MG, our results did not demonstrate a clear benefit of chronic IVIG in reducing attack frequency or steroid requirements, in line with recent reports questioning its long-term efficacy in JMG [16,17,18]. Interestingly, in the second year of follow-up in our study, patients on IVIG exhibited significantly higher QMG scores compared with those not receiving IVIG, suggesting that chronic IVIG use may reflect a marker of more severe or refractory disease rather than a direct therapeutic effect.

Steroid therapy remains a cornerstone of JMG management; however, prolonged steroid use was not associated with shorter time to remission or fewer attacks in this study. In fact, patients with extended steroid exposure tended to have higher QMG scores, consistent with prior observations that long-term corticosteroid dependence often indicates a more severe disease phenotype and exposes patients to significant side effects [19].

We further explored the role of AZP, which is commonly used as a steroid-sparing agent in pediatric MG. Our regression analysis found no significant relationship between the timing of azathioprine initiation and the number of clinical attacks, though small sample size and heterogeneity likely limited statistical power. Previous studies have suggested that the earlier introduction of azathioprine may improve long-term outcomes and reduce relapse rates, but high-quality pediatric-specific evidence remains limited [19].

Another important observation in our study was that, beyond the expected increases in QMG scores during overt clinical exacerbations, some patients demonstrated isolated score elevations despite being clinically asymptomatic. This discrepancy highlights the potential utility of systematic scale use, such as the QMG, in routine follow-up to detect subclinical deterioration and to provide a more objective assessment of treatment response. Similar findings have been reported in adult MG populations, and their relevance to pediatric disease warrants further exploration [20,21].

The presence of comorbidities was associated with a longer time to clinical stabilization, although this difference did not reach statistical significance. While attack frequency overall was comparable, patients with comorbid disorders experienced more frequent relapses before thymectomy. Antibody status showed no significant relationship with either stabilization time or the number of attacks. Notably, patients with refractory epilepsy or severe anxiety exhibited higher QMG scores and delayed stabilization, highlighting the potential influence of specific comorbidities on disease course [22,23].

Overall, our findings underscore the complexity of treatment decisions in JMG. While thymectomy appears to confer benefit, the role of chronic IVIG and prolonged corticosteroid use remains uncertain, with treatment responses varying substantially between patients. The discrepancies observed between patient-reported outcomes and objective measures such as QMG further emphasize the importance of incorporating multiple assessment tools—including PM-QOL15—in pediatric MG management. Larger, prospective multicenter studies are needed to clarify optimal treatment strategies and to validate disease-specific outcome measures in children.

### 4.1. Strengths

A key strength of this study is the application of validated disease-specific assessment tools, including the QMG and PM-QOL15 scales, which provided objective evaluation of neuromuscular status alongside patient-reported outcomes. Their combined use enabled differentiation between physical and psychological deterioration, supporting more individualized management. Additional strengths include consistent longitudinal follow-up within a single specialized center and the opportunity to evaluate less frequently reported interventions such as thymectomy and chronic IVIG in a pediatric cohort.

### 4.2. Limitations

The principal limitations include the retrospective design, small sample size, and single-center setting, which restrict statistical power and generalizability. The overrepresentation of patients undergoing thymectomy and maintenance IVIG may also introduce selection bias and limit comparability with other series. Furthermore, the relatively short follow-up period precludes conclusions regarding long-term outcomes. These limitations underscore the need for larger, multicenter prospective studies to validate these findings and refine treatment strategies in juvenile myasthenia gravis.

## 5. Conclusions

This study suggests that the systematic use of QMG and PM-QOL15 scales may enhance disease monitoring and support individualized management in juvenile myasthenia gravis. Thymectomy was associated with clinical improvement, whereas the benefits of chronic IVIG and prolonged corticosteroid use remained unclear. The influence of comorbidities on outcomes was evident but not statistically significant. Larger, prospective multicenter studies are required to validate these findings and optimize pediatric treatment strategies.

## Figures and Tables

**Table 1:** Demographic characteristics of juvenile myasthenia gravis (JMG) patients.

Patient No	Gender	Age at Dx (mo)	MGFA Class	Type of MG G/O	Transition from O to G	Transition Time from O to G	Referral Symptoms/Signs	Follow Up Duration (mo)	Antibody Type	Thymectomy	Additional Comorbidities	Tx	Tx Complications	PM-QOL15-Score	QMG Score (Mean/Median)
1	Female	111	3B	G			Nasal speech, difficulty in swallowing	72	AChR	Yes	No	PYR,CS,AZP,IVIG	Cataract	0	2.6/10
2	Male	177	4B	G			Difficulty in swallowing, respiratory arrest	48	AChR	Yes	No	PYR,CS,IVIG	No	0	3.7/1
3	Female	118	4B	G			Nasal speech, food aspiration	40	AChR	Yes	Intractable, atypical absence epilepsy	PYR,CS,AZP,IVIG	No	26	8.8/11
4	Male	156	4B	G			Nasal speech, difficulty in swallowing	48	AChR	Yes	No	PYR,CS,AZP,IVIG	No	3	3.9/2
5	Female	193	2A	G			Tiredness	48	AChR	Yes	No	PYR,CS,AZP,IVIG	No	15	8/8.5
6	Female	138	3B	O	Yes	1mo	Tiredness and pitosis	53	AChR	Yes	No	PYR,CS,AZP,IVIG	Hip fracture	23	7.5/6
7	Female	124	1	O	Yes	1 yr	Pitosis	48	AChR	No	GH def, ITP,SLE	PYR,CS,AZP	No	2	2.2/2
8	Female	143	1	O	Yes	5 yr	Ophtalmoparesis	76	AntiMUSK	No	No	PYR,CS	No	0	2.2/2
9	Male	167	3A	G			Tiredness	48	AChR	No	No	PYR,CS	No	0	4.2/3
10	Male	204	3A	G			Tiredness, weakness, diplopia	24	AChR	Yes	Anxiety Disorder	PYR,CS	No	54	9.6/11

G = Generalized; O = Ocular; Tx = Treatment; PM-QOL15 = The Pediatric Myasthenia-Quality of Life 15; PYR = pyridostigmine, CS = corticosteroid; AZP = Azathioprine; IVIG = Intravenous Immunoglobulin; Dx = diagnosis; No = number; MGFA = Myasthenia Gravis Foundation of America; yrs = years; mo = months; N/A = not applicable; AChR = Acetylcholine Receptor.

**Table 2:** Clinical characteristics of thymectomy patients.

Patient No	Thymectomy Age	Time Passed from Dx to Thymectomy (mo)	Indication for Surgery	Surgery Related Complications	Duration of Hospital Stay (Days)	Duration of Surgery (Min)	Thymus Pathology	No of Attacks Before Surgery	No of Attacks After Surgery	Follow-Up Duration After Surgery (mo)	MG Score Before Surgery	MG Score After Surgery	Medications Before Surgery	Medication at Last Visit	MG Score at Last Visit
1	128	17	Steroid dependence Frequent attacks	No	5	120	Thymic hyperplasia	4	2	56	5	0	CS 0.1 mg/kg PYR 3 mg/kg AZP 1.5 mg/kg Monthly IVIG (last 3 months)	AZP 2 mg/kg	0
2	178	8	Radiologically diagnosed thymoma	No	5	180	Thymoma	3	0	40	14	0	CS 1 mg/kg PYR 3 mg/kg	-	0
3	123	5	Resistant to treatment	No	4	135	Normal thymus	2	0	36	11	2	CS 1 mg/kg PYR 6 mg/kg AZP 1 mg/kg IVIG monthly(last 5 months)	-	0
4	168	12	Steroid dependence	No	2	120	Thymic hyperplasia	2	0	36	2	2	CS 0.2 mg/kg PYR 5 mg/kg AZP 2 mg/kg	AZP 2 mg/kg	0
5	202	8	Steroid dependence	No		120	Thymic hyperplasia	1	0	16	11	6	CS 0.1 mg/kg PYR 3 mg/kg AZP 2 mg/kg	PYR 3 mg/kg AZP 2 mg/kg	3
6	146	8	Steroid complications and dependence	No	3	120	Thymic hyperplasia	3	3	44	7	5	PYR 3 mg/kg AZP 2 mg/kg IVIG monthly(last 6 mo)	PYR 2 mg/kg AZP 2 mg/kg	3
7	229	23	Resistance to treatment	No	3	140	Thymic hyperplasia	3	0	1	4	2	PYR 2 mg/kg AZP 2 mg/kg IVIG monthly (last 6 mo)	PYR 2 mg/kg AZP 2 mg/kg	2

Dx = Diagnosis; Mo = months; Min = minutes; No = number; MG = myasthenia gravis; PYR = Pyridostigmine; AZP = Azathioprine.

**Table 3:** Follow-up characteristics of thymectomy patients.

	Mean ± S.D	Median(Min–Max)
Follow up after thymectomy	36.86 ± 17.33	42 (1–55)
Time till symptom-free	4.6 ± 5.04	3 (1–18)
Number of attacks	1.7 ± 1.57	1 (0–5)
Number of attacks before thymectomy	1.29 ± 0.95	1 (0–3)
Number of attacks after thymectomy	0.71 ± 1.25	0 (0–3)
Health quality score (PM-QOL15)	17.3 ± 19.4	15 (0–54)
QMG score (basal)	14.2 ± 2.7	14 (10–17)
QMG score before thymectomy	7.71 ± 4.39	7 (2–14)
QMG after thymectomy	2.43 ± 2.3	2 (0–6)
Time to thymectomy	7.5 ± 7.92	6 (0–23)
Med QMG score in 1st year	8.3 ± 4	6.8 (3–13)
Med QMG score in 2nd year	3.4 ± 2.4	2.7 (0–7.5)
Med QMG score in 3rd year	2.5 ± 4	1 (0–5)
Total steroid use (mo)	9.8 ± 11.2	3 (0–26)
AZP initiation time	4.2 ± 4.5	3 (1–12)

**Table 4:** QMG scores of patients on maintenance IVIG.

	Monthly IVIG				*p*
	No		Yes		
	Mean ± S.D	Median(Min–Max)	Mean ± S.D	Median(Min–Max)	
Med QMG score in 1st year	4.87 ± 1.62	5.8 (3–5.8)	7.73 ± 4.78	6.8 (1.6–13)	0.197
Med QMG score in 2nd year	1.23 ± 1.12	1.5 (0–2.2)	4.2 ± 2.04	3.6 (2–7.5)	0.049

Independent-Samples T Test.

**Table 5:** Clinical outcome related to middle/high-dose long-term steroid use.

	Middle/High Dose Steroid Use More than 3 mo				*p*
	No		Yes		
	Mean ± S.D	Median(Min–Max)	Mean ± S.D	Median(Min–Max)	
Time till symptom-free	5.5 ± 8.35	1.5 (1–18)	4 ± 1.67	3.5 (2–6)	0.745
Number of attacks	2.5 ± 1.91	2 (1–5)	1.17 ± 1.17	1 (0–3)	0.204
Number of attacks before thymectomy	2 ± 1	2 (1–3)	0.75 ± 0.5	1 (0–1)	0.078
Number of attacks after thymectomy	1 ± 1.73	0 (0–3)	0.5 ± 1	0 (0–2)	0.646

Independent-Samples T-Test.

**Table 6:** QMG scores of patients on long-term high-dose steroids.

	Steroids for More than 3 Months				*p*
	No		Yes		
	Mean ± S.D	Median(Min–Max)	Mean ± S.D	Median(Min–Max)	
Med QMG score in 1st year	5.34 ± 3.74	5.2 (1.6–13)	10.43 ± 3.23	11.5 (6.8–13)	0.076
Med QMG score in 2nd year	2.91 ± 2.09	2.2 (0–5.6)	4.23 ± 2.83	2.7 (2.5–7.5)	0.430
Number of attacks after thymectomy	0.5 ± 1	0 (0–2)	1 ± 1.73	0 (0–3)	0.646

Independent-Samples T-Test.

## Data Availability

The original contributions presented in this study are included in the article. Further inquiries can be directed to the corresponding author.

## Abbreviations

AZP	Azathioprine
IVIG	Intravenous Immunoglobulin
JMG	Juvenile Myasthenia Gravis
MGFA	Myasthenia Gravis Foundation of America
PM-QOL15	Pediatric Myasthenia-Quality of Life 15
QMGS	Quantitative Myasthenia Gravis Scoring
